# Polycystic Ovary Syndrome-Associated Acne: The Interplay of Hyperandrogenism, Insulin Resistance, and Therapeutic Strategies

**DOI:** 10.7759/cureus.98103

**Published:** 2025-11-29

**Authors:** Eftichia Damoulaki, Dimos Sioutis, Vaia Sarli, Eutychios Trakakis, George Mastorakos, Alexander Katoulis, Konstantinos Kastrinakis, Christos Koratzanis, Nikolaos Machairiotis, Periklis Panagopoulos, Chrysi Christodoulaki

**Affiliations:** 1 Third Department of Obstetrics and Gynecology, Attikon University Hospital, National and Kapodistrian University of Athens, Athens, GRC; 2 Division of Endocrinology, Diabetes and Metabolism, Aretaieion University Hospital, National and Kapodistrian University of Athens, Athens, GRC; 3 Second Department of Dermatology and Venereology, Attikon University Hospital, National and Kapodistrian University of Athens, Athens, GRC; 4 Department of Obstetrics and Gynecology, General Hospital of Chania, Chania, GRC

**Keywords:** acne vulgaris, combined oral contraceptives, hyperandrogenism, insulin resistance, polycystic ovary syndrome (pcos), sebum production

## Abstract

Acne vulgaris is one of the most prevalent dermatologic disorders in adolescent and adult women. Beyond classic pathogenic factors, such as sebaceous hypersecretion, follicular hyperkeratinization, *Cutibacterium acnes* overgrowth, and inflammation, endocrine drivers are pivotal. Polycystic ovary syndrome (PCOS) is common across reproductive ages and frequently presents with dermatologic manifestations, notably acne, through mechanisms dominated by hyperandrogenism and compounded by insulin resistance (IR). This narrative review synthesizes current evidence linking PCOS with acne, explains the interrelated roles of hyperandrogenism and IR, and outlines evidence-based management strategies tailored to women with PCOS.

The Rotterdam diagnostic framework remains the globally favored standard for PCOS, emphasizing combinations of oligo-/anovulation, clinical/biochemical hyperandrogenism, and polycystic ovarian morphology. PCOS pathophysiology involves dysregulation of the hypothalamic-pituitary-ovarian axis with increased gonadotropin-releasing hormone pulsatility, elevated luteinizing hormone, and suppression of follicle-stimulating hormone, promoting thecal androgen excess and impaired folliculogenesis. IR, prevalent in PCOS, augments ovarian/adrenal androgen synthesis and lowers sex hormone-binding globulin levels, increasing free testosterone and sebum production; IGF-1 signaling further amplifies sebogenesis and follicular hyperkeratinization. Emerging data implicate genetic/epigenetic determinants, endocrine-disrupting chemicals, gut microbiome dysbiosis, and endoplasmic reticulum stress as modulators of the PCOS-acne phenotype. Epidemiologic studies and meta-analyses report higher acne prevalence and severity in PCOS, particularly among adolescents.

First-line therapy includes combined oral contraceptive pills with antiandrogenic progestins; spironolactone is an effective adjunct. Where indicated, metformin addresses IR and may improve acne and ovulatory function. Judicious use of standard acne modalities such as topical retinoids, benzoyl peroxide, and oral/topical antibiotics remains essential; oral isotretinoin is reserved for refractory disease with careful risk management. PCOS-associated acne reflects converging endocrine and metabolic disturbances. Integrating androgen-targeted therapy, IR mitigation, and guideline-based acne care provides the most durable control. Future research should refine phenotype-directed treatment and clarify the contributions of microbiome, environmental exposures, and cellular stress pathways.

## Introduction and background

Acne (acne vulgaris) comprises the majority of skin conditions in the general population and is one of the most common skin diseases, notably in adolescent and young adult females [1]. Outlining acne’s comprehensive picture, it is described as a dermatological disorder triggered by excess oil, clogged pores, and bacteria that naturally live on the skin. Under certain circumstances, such as immoderate sebum production and extreme dead skin cell shedding, the tiny skin pores get blocked, attracting microbes, enabling them to flourish and cause spots/blemishes and inflammation in the skin follicles [2,3]. These blemishes include whiteheads, blackheads, pimples, and even cysts. If acne is left untreated or not given the appropriate medical attention, it can cause chronic inflammation, secondary infections, scarring, discoloration, and hyperpigmentation, leading to low self-esteem, anxiety, depression, and poor quality of life [4].

It is imperative to understand that acne can be either a primary disease in most adolescents or a case of comorbidity mainly in adults. As a primary skin disease, it is considered to be a potential intrinsic process during puberty rather than an outcome due to undetected systemic disorders [5]. In particular, the sebaceous glands are activated by elevated androgen levels, which are linked to natural hormonal changes, inducing an excessive secretion of sebum. This fact, in combination with follicular hyperkeratosis, results in the obstruction of hair follicles, creating microcomedones [4]. Given these conditions, the proliferation of *Cutibacterium acnes* (a commensal bacterium of the natural skin microbiota) is stimulated, leading to localized inflammation and an expected inflammatory reaction, such as swelling, redness, discomfort, pain, and blisters [4,6].

While hormonal variations are less frequent in older age groups, contributing to lower acne prevalence in adult individuals, the underlying pathophysiology remains the same. The difference resides in the pathogenesis of this skin condition. Focusing on adult women, the etiology of acne vulgaris has its roots in genetics, diet, treatments, cosmetics, stress, and other reasons that can cause hormonal imbalances, as well as disruption of the cutaneous microbiome [7,8]. Polycystic ovarian syndrome (PCOS) and insulin resistance, in addition to being interrelated, also constitute a significant integrative mechanism in acne development [9].

## Review

It is noteworthy that even though PCOS has been studied for at least 100 years, a unified definition of the syndrome does not exist to date. This is attributed to the phenotypic diversity of women with PCOS. However, the predominant manifestations are oligomenorrhea or amenorrhea, infertility, polycystic ovarian morphology (PCOM), masculinization, hirsutism, weight gain, and oily skin [10]. The display of the symptoms differs between individuals, regarding both severity and combination; there are consequential health effects, including insulin resistance, type II diabetes mellitus, metabolic syndrome, dyslipidemia, hypertension, atherosclerosis, endometrial dysfunction, androgenic alopecia, acanthosis nigricans as well as a higher risk of recurrent miscarriages, preterm birth, endometrial hyperplasia or carcinoma, even anxiety and depression [11-16]. The scientific community primarily applies three main sets of criteria in order to identify whether PCOS occurs or not: the National Institutes of Health Criteria (NIH, 1990), the Rotterdam Criteria (2003), and the Androgen Excess and PCOS Society Criteria (AES, 2006) [17]. A clinical diagnosis of PCOS based on the Rotterdam Criteria necessitates the presence of at least two of the following three symptoms: oligomenorrhea or amenorrhea (caused by oligo-ovulation or anovulation, respectively), hyperandrogenemia (elevated androgen levels in the blood that are not inevitably related to the presence of corresponding clinical outcome) or hyperandrogenism (presence of androgenic features due to increased androgen levels) and PCOM evident on ultrasound [10,18]. The Rotterdam Criteria remain the most accepted and comprehensive diagnostic framework for PCOS globally, and require a detailed clinical, biochemical, and imaging evaluation. Concerning the other two diagnostic sets, the NIH Criteria do not incorporate ultrasound as an essential diagnostic method and focus on chronic amenorrhea/oligomenorrhea and hyperandrogenemia/hyperandrogenism, whereas the AES Criteria put hyperandrogenemia/hyperandrogenism at the forefront and amenorrhea or PCOM as a secondary consideration [17-19].

The diagnostic assessments conducted to confirm PCOS encompass several tests, starting with collecting an in-depth menstrual history in conjunction with biochemical evaluations of gonadotropins and estradiol levels (on Days 2-5 of the menstrual cycle) to determine the presence of oligomenorrhea or amenorrhea [20]. Clinical findings are also crucial, such as excess hair growth in androgen-sensitive areas, acne, and androgenic alopecia, since they could indicate hyperandrogenism, establishing biochemical tests of serum androgens as a mandatory component of the diagnostic process, specifically the measurement of total and free testosterone, as well as dehydroepiandrosterone sulfate (DHEAS) levels [21]. Furthermore, transvaginal/transabdominal ultrasonography should be undertaken to ascertain PCOM (PCOM: either an ovary with >20 follicles, which are little fluid-filled sacs with immature eggs and have a diameter of 2-9 mm each, or an elevated ovarian volume over 10 cm³) [20]. It should be emphasized that other causes that may affect the diagnosis must be ruled out by adding thyroid function and non-classical congenital adrenal hyperplasia tests, conditions that mimic PCOS symptoms.

As observed in the majority of hormonal syndromes, PCOS epidemiology relies strongly on different age groups and populations, leading to a variable prevalence of females affected by this condition. In the case of PCOS, the broad range also derives from the alternative application of the aforementioned diagnostic methods. Therefore, scientific studies demonstrate that PCOS prevalence in puberty varies from 3% to 11%, in adult women (of reproductive age) is estimated from 6-18%, while the worldwide prevalence is calculated to be 9.2%. The highest rates are reported in adult women aged 20-29 years, with an upward trend in the adolescents aged 15-19 years [17, 22-24]. Nevertheless, it must be acknowledged that defining PCOS clinically in adolescents is often challenging, because features of normal puberty can be mistakenly not considered as diagnostic criteria. PCOS is among the most frequent endocrine disorders, and its global prevalence is detected to have nearly doubled since 1990, due to several environmental factors, higher obesity rates, aging societies, and enhanced diagnostic assessment [22,24].

The mechanisms involved in PCOS pathophysiology stimulate ovaries to produce an increased concentration of androgens, disrupting the overall hormonal balance in the female reproductive system and impeding follicle ovulation. A variety of genetic factors appear to be responsible for this hormonal abnormality, for example, several gene mutations on loci related to steroidogenesis, gonadotropin regulation, and insulin resistance, affecting the physiological function of steroidogenic enzymes and androgen receptors, along with epigenetic modifications, which further modulate the expression of these genes [10, 25-28]. Likewise, multiple environmental contributors have been found to influence genetic and hormonal pathways associated with PCOS, for instance, lifestyle factors and various endocrine-disrupting chemicals (EDCs) [25,29,30]. Moreover, a number of studies researching the relation between PCOS and gut microbiome dysbiosis or PCOS and endoplasmic reticulum (ER) stress, and even PCOS and prenatal androgen exposure have garnered an increasing scientific interest. Regarding PCOS and gut microbiome, it has been demonstrated that changes in the composition of gut bacteria (dysbiosis) have been closely associated with an increased release of endotoxins (e.g., lipopolysaccharides), triggering systemic inflammation, which deteriorates insulin resistance and PCOS [31,32]. Proceeding to the correlation between ER stress and PCOS, it is a vicious cycle within the syndrome - PCOS activates ER stress in ovarian granulosa cells through mechanisms like hyperandrogenism, insulin resistance, oxidative stress, and inflammation. Then, ER stress reinforces the impact of PCOS through cell dysfunction, apoptosis, and ovarian fibrosis [33,34]. In addition, studies in human and animal models show a crucial relation between excess prenatal androgen exposure and PCOS phenotype in female adulthood (hyperandrogenism, ovulatory dysfunction, and metabolic abnormalities). This is caused by disrupting the development of the hypothalamic-pituitary-ovarian axis, leading to alterations in gene expression and epigenetic regulation [35,36].

Normally, follicle development in the ovary is regulated by follicle-stimulating hormone (FSH), but in PCOS, this hormone remains at low levels, leading to the arrest of follicular maturation, a condition where follicles stop halfway through their development and turn into cysts. As a result, ovulation is absent since no mature egg is produced or released (oligo-anovulation). In parallel, the excess amount of luteinizing hormone (LH), together with the elevated levels of insulin occurring in the syndrome, induces and maintains the overproduction of androgens. The etiology underlying these hormonal disruptions is based on the dysregulation of the hypothalamic-pituitary-ovarian (HPO) axis [37]. HPO axis’ altered regulation subsequently disrupts the normal pattern of gonadotropin secretion, initiating the elevation in the frequency and amplitude of gonadotropin-releasing hormone (GnRH) pulses. This increase selectively stimulates LH secretion over FSH, contributing to an enhanced LH/FSH ratio, which is usually used as a biochemical hallmark of PCOS [38]. The excess amount of LH triggers ovarian theca cells to overproduce androgens, leading to hyperandrogenism and disrupting once again the follicle maturation and the ovulation. Ovarian theca cells are also stimulated by increased insulin levels caused by PCOS, elevating the androgen production, as well as suppressing the hepatic production of sex hormone-binding globulin (SHBG). The inhibition of SHBG, in turn, increases the bioavailable testosterone. Additionally, the excess amount of insulin can also modulate follicular development and ovulation via insulin-like growth factors [39].

Acne has steadily been found to constitute one of the main symptoms of PCOS, both in adolescents and adult women. Its overall prevalence is estimated to be 40-70% in the female population with PCOS (at least twice the prevalence of all women with acne, as it is already mentioned), with the higher rates reported in adolescents [40-42]. In the case of PCOS, the primary reason for acne is hyperandrogenism, which is considered the hallmark of the syndrome. Increased androgens, namely testosterone and its potent derivative dihydrotestosterone (DHT), exhibit the ability to stimulate in a direct manner the sebaceous glands in the skin, elevating sebum production, leading to an “environment” which promotes acne development by promoting the creation of clogs and microcomedones, while they can also cause alterations in keratinization within the follicle lining by damaging the physiological process of dead skin cells shedding and further inducing pore blockage. Under these circumstances, *Cutibacterium acnes* initiates its colonization on the skin, facilitated by systemic inflammatory mediators, which are often increased in PCOS, triggering inflammation, causing papules and pustules. This chronic, cutaneous inflammatory response usually impairs normal tissue healing, resulting in acne persistence and possible skin scarring [25].

Over the past decade, multiple studies and reviews investigated the link between PCOS and acne by examining the frequency of acne in the female population with PCOS and healthy individuals, respectively. A comprehensive meta-analysis, which is comprised 240,213 women with PCOS and over 1,900,000 control subjects revealed that the overall acne prevalence in the female population with the syndrome was 43% (42% in adult women and 59% in adolescents), while in healthy women, the overall prevalence was estimated at 21%, highlighting that acne prevalence depended on region (East Asian women showed the highest prevalence), as well as on the diagnostic framework of PCOS [42]. A review reported a similar acne pattern in women with PCOS by reviewing 18 studies, mentioning a significantly increased prevalence compared to healthy women. A prospective study with 212 acne patients demonstrated that 65.6% of them had been further diagnosed with PCOS. Moreover, PCOS-related acne patients showed a higher chance of having comorbid hirsutism or/and androgenic alopecia than non-PCOS-related acne patients; their main testosterone levels were notably higher, while their LH/FSH ratio surpassed 1 in over 70% of cases (a twofold higher percentage compared to non-PCOS-related acne patients). Furthermore, the study highlighted a relation between hypertestosteronemia-hyperprolactinemia and severe acne in women with PCOS [40,41]. However, there are a few relatively older studies that question or even dispute the direct link between acne and PCOS, based on the failure of many researchers to give an in-depth explanation of how serum androgen levels are correlated with acne in PCOS women. In addition, other studies suggest that acne should be considered as a less reliable marker for PCOS, because it is shown to be dependent on various factors, like hirsutism and lifestyle [43,44].

A supplementary pathophysiological mechanism involved in the development of acne in women with PCOS is insulin resistance [45,46]. Insulin resistance is a metabolic disorder in which glucose uptake by various tissues (e.g., skeletal muscles, liver, and adipose tissue) is less effective, regardless of normal or even elevated insulin levels in the blood, since insulin’s main role is to promote this process. As a consequence, pancreatic beta cells, in order to surmount the attenuated tissue response and maintain glucose homeostasis (at least in early stages), begin to overproduce insulin, leading to hyperinsulinemia. Gradually, if beta cells do not succeed in restoring homeostasis, insulin resistance and hyperinsulinemia can turn to hyperglycemia and type II diabetes, while they are related to other metabolic disorders like hypertension and dyslipidemia [47,48]. In the case of insulin resistance, its receptors on the cell membrane fail to bind with insulin and trigger its signaling cascade, facilitating, eventually, glucose uptake via glucose transporter type 4 (GLUT4) [49]. The impairment of insulin’s binding with its receptors occurs through several signaling routes, such as reduced insulin receptor tyrosine kinase activity, decreased phosphorylation of insulin receptor substrate-1 (IRS-1), dysregulated downstream pathways (e.g., PI3K/Akt), chronic inflammation, and oxidative stress [50].

PCOS and insulin resistance are interconnected and mutually reliant. Firstly, hyperandrogenism caused by PCOS can disrupt physiological insulin signaling reactions through the excess androgens, which dysregulate insulin receptor function and, as a result, the glucose uptake in peripheral tissues [39]. Respectively, high levels of glucose, due to insulin resistance, are combined with increased levels of LH because of PCOS, elevating ovarian androgen production and inhibiting hepatic synthesis of SHBG, resulting in anovulation [39,51]. The essential point in the connection of insulin resistance (as an outcome of PCOS) and acne is that overproduction of insulin leads to increasing levels of insulin-like growth factor 1 (IGF-1). This IGF-1 elevation further enhances androgen synthesis in the ovaries, as well as in the adrenal glands, intensifying hyperandrogenism and increasing sebum production, leading to a pro-acneic state [52,53].

Recent literature indicates a great association between insulin resistance in PCOS and acne, due to hyperandrogenism and other hormonal/metabolic disturbances, caused by the syndrome [53,54]. A retrospective study selected data from 305 women with PCOS and demonstrated that 56.4% of them had acne, while elevated insulin resistance markers and dehydroepiandrosterone sulfate (DHEAS) levels were highly correlated with both the presence and severity of this skin condition [55]. Also, a cross-sectional study on 112 women with PCOS (of which 39% were diagnosed with insulin resistance) reported that 53.57% of insulin-resistant patients had acne, highlighting the fact that acne alone was not found to be statistically different between insulin-resistant and non-insulin-resistant women [56].

The primary medical strategy regarding the treatment of acne in women with PCOS consists of drugs targeting androgen overproduction and binding to their receptors, as well as topical therapies and antibiotics. As first-line therapy, combined oral contraceptive pills (COCPs), a combination of estrogens and antiandrogenic progestins (drospirenone/cyproterone acetate), are administered in order to reduce circulating androgens and induce SHBG, which can lower free testosterone levels, resulting in acne suppression [57]. Complementary to COCPs, one more substance that is widely used to treat PCOS-associated acne is spironolactone, an effective androgen receptor antagonist (and also a 5α-reductase inhibitor), which has the ability to reduce the impact of androgens on sebaceous glands (and sebum production) by blocking androgen receptors in the skin, leading to amelioration of acne [58]. Limitations (e.g., migraine, thrombophilia, heavy smoking) and side effects (e.g., teratogenesis) of those therapies ought to be taken into consideration, and patients need to be regularly monitored. Oral isotretinoin is used as an alternative therapeutic approach when COCPs are not a safe option. Isotretinoin induces expression of apoptosis-promoting factors, such as p53, resulting in a decrease in androgens, therefore also in the downregulation of sebum, testosterone, and insulin production. Nevertheless, additional research is needed in order to elucidate its side effects (e.g., dyslipidemia) [59]. These systemic hormonal therapies are most of the time co-administered with various topical treatments like retinoids, salicylic acid, and benzoyl peroxide, along with antibiotics (both oral and topical), such as clindamycin (topical administration), doxycycline, or minocycline (mainly oral administration) [60,61]. Furthermore, in the case of comorbidity of PCOS, insulin resistance, and acne, metformin is used (alone or in combination with other treatments) as an insulin sensitizer, reducing androgen levels, enhancing ovarian function, and significantly improving acne [62,63].

At present, although scientists have a substantial understanding of the pathophysiology of PCOS, as well as its diagnosis and treatment, there are still crucial questions that have not been fully answered regarding acne, specifically. For example, recent studies have detected additional factors contributing to PCOS-associated acne, like genetic predispositions, which can modulate androgen receptor sensitivity and sebaceous gland responsiveness [64]. Moreover, metabolic comorbidities as obesity and insulin resistance appear to have further impact on acne development in women with PCOS and are under investigation. Of particular significance is the fact that despite PCOS being a highly prevalent condition in both adolescents and adult women, leading to notable metabolic disorders and infertility, it often remains underdiagnosed due to low awareness and unharmonized diagnostic criteria, limiting early detection. This observation plays a key role in PCOS treatment outcomes, especially in acne, which may vary from mild to severe and is often persistent or even resistant to treatment.

## Conclusions

Acne vulgaris is not merely a cosmetic concern but a frequent and often distressing manifestation of PCOS. Its pathogenesis in this context is multifactorial, with hyperandrogenism and insulin resistance serving as the cornerstone mechanisms that exacerbate sebaceous activity, follicular keratinization, and inflammatory responses. The high prevalence of acne among women with PCOS, particularly adolescents, underlines its clinical relevance as both a diagnostic clue and a therapeutic challenge.

Effective management requires a multidisciplinary approach that combines endocrine regulation with standard dermatological care. Hormonal interventions, such as combined oral contraceptives and antiandrogens, remain the cornerstone of treatment, while insulin sensitizers like metformin play an increasingly important role, particularly in women with metabolic comorbidities. Conventional topical and systemic acne therapies continue to be essential adjuncts, especially in patients with moderate-to-severe disease.

Despite advances, significant gaps remain in understanding the genetic, metabolic, and environmental modulators of PCOS-associated acne. Greater awareness, earlier recognition, and phenotype-specific strategies are needed to optimize patient outcomes. Future research should focus on precision medicine approaches that integrate endocrine, metabolic, microbiome, and lifestyle factors to deliver more individualized and durable solutions for women affected by both PCOS and acne.
